# Brain Neural Activity Patterns in an Animal Model of Antidepressant-Induced Manic Episodes

**DOI:** 10.3389/fnbeh.2021.771975

**Published:** 2022-02-16

**Authors:** Min Chen, Guangdong Chen, Hongjun Tian, Guangqian Dou, Tao Fang, Ziyao Cai, Langlang Cheng, Suling Chen, Ce Chen, Jing Ping, Xiaodong Lin, Chunmian Chen, Jingjing Zhu, Feifei Zhao, Chuanxin Liu, Weihua Yue, Xueqin Song, Chuanjun Zhuo

**Affiliations:** ^1^Micro-imaging Center of Psychiatric Disorder, Institute of Mental Health, Jining Medical University, Jining, China; ^2^Center of Psychiatric Animal Model, Institute of Mental Health, Wenzhou Seventh Peoples Hospital, Wenzhou, China; ^3^Department of Psychiatry Medical Center, Wenzhou Seventh Peoples Hospital, Wenzhou, China; ^4^Department of Clinical Laboratory, Wenzhou Seventh Peoples Hospital, Wenzhou, China; ^5^Key Laboratory of Real Time Tracing of Brain Circuits in Psychiatry and Neurology (RTBNP_Lab), Nankai University Affiliated Tianjin Fourth Center Hospital, Tianjin Fourth Center Hospital, Tianjin, China; ^6^PKU-IDG/McGovern Institute for Brain Research, Peking University, Beijing, China; ^7^Department of Psychiatry, The First Affiliated Hospital of Zhengzhou University, Zhengzhou, China

**Keywords:** bipolar disorder, brain calcium activity, phase switching, pre-pulse inhibition, symptoms’expression

## Abstract

**Background**: In the treatment of patients with bipolar disorder (BP), antidepressant-induced mania is usually observed. The rate of phase switching (from depressive to manic) in these patients exceeds 22%. The exploration of brain activity patterns during an antidepressant-induced manic phase may aid the development of strategies to reduce the phase-switching rate. The use of a murine model to explore brain activity patterns in depressive and manic phases can help us to understandthe pathological features of BP. The novel object recognition preference ratio is used to assess cognitive ability in such models.

**Objective**: To investigate brain Ca^2+^ activity and behavioral expression in the depressive and manic phases in the same murine model, to aid understanding of brain activity patterns in phase switching in BP.

**Methods**: *In vivo* two-photon imaging was used to observe brain activity alterations in a murine model in which induce depressive-like and manic-like behaviors were induced sequentially. The immobility time was used to assess depressive-like symptoms and the total distance traveled was used to assess manic-like symptoms.

**Results**: *In vivo* two-photon imaging revealed significantly reduced brain Ca^2+^ activity in temporal cortex pyramidal neurons in the depressive phase in mice exposed to chronic unpredictable mild stress compared with naïve controls. The brain Ca^2+^ activity correlated negatively with the novel object recognition preference ratio within the immobility time. Significantly increased brain Ca^2+^ activity was observed in the ketamine-induced manic phase. However, this activity did not correlate with the total distance traveled. The novel object recognition preference ratio correlated negatively with the total distance traveled in the manic phase.

## Introduction

Bipolar disorder (BP) is a chronic psychiatric disorder affecting more than 1–4% of the global population (Grande et al., 2016). Although its clinical expression is usually associated with the disturbance of brain activity, the brain functional alterations occurring during depressive and manic episodes in patients with BP have not been described precisely. In addition, antidepressant use may induce manic episodes in at least 20% of patients with BP. Hypotheses regarding specific disturbances of brain neural activity, circuits, and networks in patients with BP have been supported by macro-neuroimaging studies, especially those in which functional magnetic resonance imaging (MRI) and electroencephalography (EEG) have been used (Berk, 2009; Berk et al., 2011, 2014; Fries et al., 2012; Schneider et al., 2012). Unexpectedly, these studies have demonstrated that brain alterations and cognitive impairment are consistently associated not with the timing of first BP episodes (whether depressive or manic), but with repeated manic or depressive episodes (El-Badri et al., 2001; Strakowski et al., 2002; Lyoo et al., 2006; Robinson and Ferrier, 2006).

Due to the limitations of conditions and lack of reference knowledge, the trajectories of dynamic alterations in brain functional activity, cognition, and behavior have rarely been observed in the same patient or animal model. The exploration of brain activity alterations in the same animal model during first the depressive phase and then the manic phase can provide evidence aiding the understanding of the pathological features of BP. Such models are particularly suitable for the examination of brain activity features in the antidepressant-induced manic phase of BP, as they often involve the use of ketamine to induce mania (Liu et al., 2018; Bhatt et al., 2021; Gao et al., 2021). Ketamine has an antidepressant effect and can rapidly alleviate serious depressive symptoms (Meshkat et al., 2021; Muscat et al., 2021).

In this pilot study, we used *in vivo* two-photon imaging to examine brain activity features in the depressive and manic phases in the same murine model of BP. As depressive episodes usually precede manic episodes in clinical practice, we modeled depressive episodes using chronic unpredictable mild stress (CUMS), followed by the induction of the manic phase with ketamine (thus representing antidepressant-induced mania in BP) in the same animals. We proposed two hypotheses: (1) that brain Ca^2+^ activity patterns in the depressive and manic phases would differ, and (2) that brain Ca^2+^ alterations would be associated with cognitive and behavioral performance.

## Materials and Methods

### Animals and Experimental Design

Male C57BL/6 mice from multiple litters (Japan SLC Inc., Shizuoka, Japan) were used in this study. Eight groups of six mice each were housed in group cages. The experiments were performed using polycarbonate cages (18 × 30 × 17 cm) designed for “24 h home-cage activity and social behavior monitoring” (Ohara Co. Ltd., Tokyo, Japan). Four mice were kept in each cage. The mice were supplied with Palsoft paper bedding (Oriental Yeast, Tokyo, Japan). The animal room was maintained at 50%±10% humidity and a temperature of 23±2°C under a 12-h light/12-h dark cycle (lights on from 06:00–18:00). The mice were allowed free access to CE-2 food (CLEA Japan, Tokyo, Japan) and water. The mice were maintained and all experiments were performed in accordance with guidelines provided by National Institute of Mental Health^1^, and all procedures were approved by the Committee for Animal Care and Use of Tianjin Medical University Affiliated Tianjin Fourth Centre Hospital (IRB-no. 801758).

First, we established the depressive phase by exposing the mice to CUMS using a standard protocol to provoke depressive behavior. One day later, according to a BP model designed to mimic a protocol for mania prevention (Banwari et al., 2015; Bhatt et al., 2021; Gao et al., 2021), we initiated a course of daily intraperitoneal injection of ketamine (25 mg/kg) to provoke manic behavior (Liu et al., 2018; Bhatt et al., 2021; Gao et al., 2021). Assessments were performed after the establishment of each phase.

### Stereotaxic Injection

Anesthesia was induced with 1.25% avertin, the scalp was incised and locally sterilized, and the periosteal tissue was removed. A stereotaxic instrument (RWD, China) was used to identify the hindlimb region of the primary somatosensory cortex (S1HL; about 0.5 mm anterior to and 1.5 mm lateral of bregma). An injection hole was created on the cranium with a high-speed microdrill (OmniDrill35; WPI, USA), and a glass microelectrode connected to an ultra-micro-injection pump (Nanoliter 2010; WPI) was used to inject 80 nl AAV2/1-hSyn-GCaMP6 s or 150 nl AAV2/1-hSyn-DIO-GCaMP6s (>1 × 10^13^ gene copies/ml; University of Pennsylvania Gene Therapy Program Vector Core) into the fifth cortical layer at a 60° angle to avoid imaging site damage. The glass electrode was kept in the brain tissue for a total of 5 min.

### *In vivo* Two-Photon Calcium Imaging

Three weeks after stereotaxic injection, the mice were anesthetized with 1.25% avertin, the skull was exposed, and two metal bars were attached to the rostral and caudal portions of the skull, respectively, with glue (Loctite 401) and dental cement to ensure head restraint during imaging. One day later, a high-speed microdrill was used to create an imaging window above S1HL. A glass coverslip was applied to the window using Vetbond tissue adhesive (3M, USA).

*In vivo* calcium imaging was performed on awake mice under head restraint using a 920-nm excitation laser with a water-immersed objective (20×, 1.1 numerical aperture; Zeiss, Germany). Under an LSM780 two-photon microscope (Zeiss), calcium activity was recorded at 2 Hz for 2.5 min at the apical tufts (0–80 μm from pia), vasoactive intestinal polypeptide somas and axons (200–300 μm from pia), somatostatin somas and axons (400–500 μm from pia), and layer 5 pyramidal neuron somas (600–650 μm from pia). These regions of interest were defined manually. Calcium-signal time series were corrected using the TurboReg plugin for ImageJ software (National Institutes of Health, Bethesda, MD, USA). Mean pixel values were averaged to obtain fluorescent (*F*) values, normalized as (Δ*F − F*_0_)/*F*_0_, where *F*_0_ (the baseline value) was the average obtained during the first 10% of recording. Total integrated calcium values were calculated by summing Δ*F*/*F*_0_ for the entire time series. Calcium spikes were defined as ≥3-standard-deviation increases.

### Behavioral Assays

The animals were subjected to a sucrose preference test as described previously (Can et al., 2012; Sharma et al., 2016), followed by a prepulse inhibition (PPI) test adapted for the quantification of sensory gating function (Feldcamp et al., 2017). After acclimation of the mice in a sound-isolating chamber with 65 dB background noise, a 75-dB prepulse (PP) was applied for 20 ms, followed 100 ms later by a 40-ms 120-dB startle stimulus (PA). The mice completed three trials with intervening intervals of 30 s. Scores were averaged and the PPI ratio was calculated as (PA − PP)/PA × 100%.

### Statistical Analysis

Data are presented as means ± standard errors of the mean unless specified otherwise. Data were compared using a one-way analysis of variance and *post hoc* Tukey tests. Data analysis and figure plotting were performed with the GraphPad Prism software (version 8.0).

## Results

### CUMS and Ketamine Exposure Evoked Abnormal Cortical Transmission and Behavior

**Figure 1:**
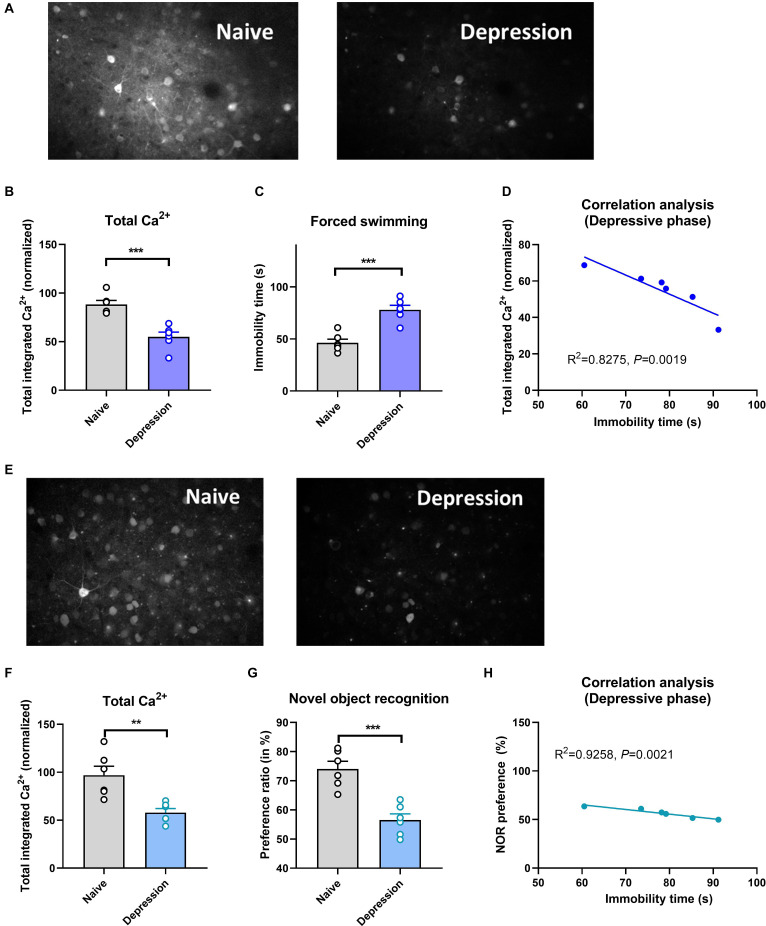
Brain Ca^2+^ activity and behavioral index alterations in mice with BPD exposed to CUMS and controls. **Note**: Compared with the naïve group (six mice, group-housed), mice in the depressive phase (*n* = 6, group-housed) showed significantly decreased normalized integrated Ca^2+^ activity (96.28 ± 11.17 vs. 53.00 ± 8.25, *P* < 0.001, **A,B**) and more immobility time (86.52 ± 9.22 vs. 47.67 ± 5.31, **C**), and these variables correlated with each other **(D)**; mice in the depressive phase also showed Ca^2+^ hypoactivity in the prefrontal cortex **(E,F)**, and had poorer novel object recognition preference ratios **(G)**. The preference ratio (pre-pause index) also correlated with the immobility time **(H)**. **P < 0.01; ***P < 0.001, vs. the naïve group.

**Figure 2:**
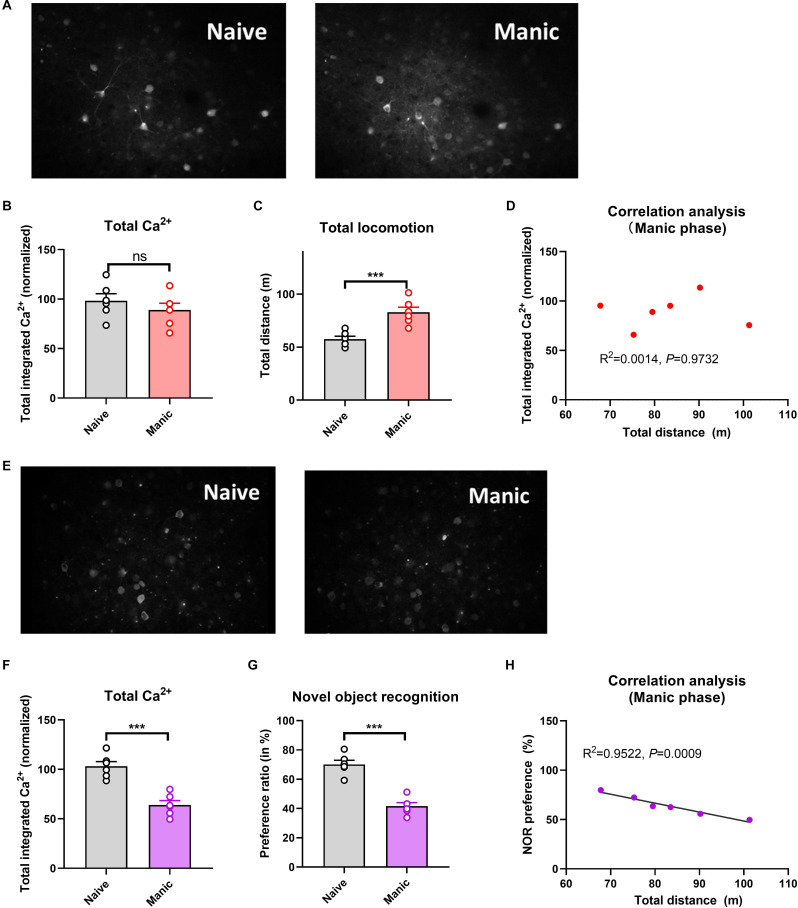
Brain Ca^2+^ activity and behavioral index alterations in mice with BPD exposed to ketamine and controls. **Note**: Compared with the naïve group (six mice, group-housed), mice in the manic phase (*n* = 6, group-housed) showed similar normalized integrated Ca^2+^ activity (96.89 ± 13.00 vs. 92.18 ± 10.44, **A,B**) but significantly more total locomotion (53.11 ± 9.25 vs. 79.66 ± 7.77 m, **C**); mice in the manic phase also showed Ca^2+^ hypoactivity in the prefrontal cortex **(E,F)**, and had poorer novel object recognition preference ratios **(G)**. The preference ratio, but not the normalized integrated Ca^2+^ activity, correlated with the total distance traveled **(D,H)**. ***P < 0.001, vs. the naïve group; ns, not significant.

Compared with naïve controls, mice exposed to CUMS had significantly longer immobility times in the depressive phase (*P* < 0.001; Figure 1C) and significantly longer total distances traveled in the manic phase (*P* < 0.001; Figure 2C). Two-photon calcium imaging demonstrated significantly decreased Ca^2+^ activity in temporal cortex (TPC) pyramidal neurons in the depressive phase, and significantly increased activity in the manic phase (Figures 1A,B, 2A,B). Compared with the naive controls, Ca^2+^ hypoactivity was observed in the prefrontal cortex in the depressive and manic phases in mice exposed to CUMS followed by ketamine (Figures 1E,F, 2E,F). The normalized total integrated calcium values correlated significantly with immobility times in the depressive phase, but not the manic phase (both *P* < 0.001; Figures 1D, 2B), due primarily to decreased calcium spike frequencies (*P* < 0.001; Figures 2C,D). In both phases, exposed mice had poorer novel object recognition preference ratios than did controls (both *P* < 0.001; Figures 1G, 2G). This ratio correlated negatively with the immobility time in the depressive phase and with the total distance traveled in the manic phase (Figures 1H, 2H). Thus, mice exposed to CUMS and ketamine showed dual phenotypes of depressive and manic behavior largely consistent with the behavioral expression of patients with BP, reflecting the successful establishment of the BP model with an antidepressant-induced manic phase. They exhibited impaired cognitive ability in the depressive and manic phases, which was more severe in the manic phase.

### Relationships Among Brain Ca^2+^ Activity, Symptom Severity, and Cognitive Impairment

Brain Ca^2+^ activity correlated negatively with the severity of depressive symptoms (as reflected by the immobility time; Figure 1D). A similar, but nonsignificant trend was observed for the severity of manic behavior (Figure 2D). Cognitive impairment was observed in the depressive and manic phases but was more severe in the manic phase.

## Discussion

This pilot microimaging study conducted with a murine model of BP with an antidepressant-induced manic phase yielded three main findings. First, compared with naïve controls, exposed mice showed significantly decreased brain Ca^2+^ activity in the depressive phase, which is consistent with macro-imaging findings suggesting that the disruption of the emotional network contributes to mood dysregulation in patients with BP (Drevets et al., 1997; Kempton et al., 2008; Bora et al., 2010; Ellison-Wright and Bullmore, 2010; Nortje et al., 2013; Hibar et al., 2018; Fries et al., 2019; Matsuo et al., 2019; Harrison et al., 2021). Second, brain Ca^2+^ activity did not increase in the manic phase, inconsistent with some previous findings (Ghedim et al., 2012; Ettenberg et al., 2020; Gao et al., 2021). This difference could be explained by the design of our murine model, in which the mice received a “double hit” due to the use of CUMS followed 1 day later by ketamine, in contrast to models of ketamine-induced manic behavior alone. The mice may not have had sufficient time to recover from the brain alterations caused by CUMS before ketamine injection, leading to the lack of an increase in brain Ca^2+^ activity in the manic phase. Third, cognitive impairment was a common feature of the depressive and manic phases and correlated with the severity of depressive and manic symptoms, but it was more severe in the manic phase.

Numerous reports, especially those on macro-imaging (particularly MRI) studies, describe decreased brain activity in the TPC in the depressive and manic phases of BP. For example, Cerullo et al. (2014) reported decreased middle temporal gyrus activation during the depressive phase in patients with BP and those with unipolar depression. Xiao et al. (2019) observed reduced cortical regional homogeneity (ReHo) in the superior temporal gyrus and increased ReHo in the cerebellum in the manic phase in patients with BP relative to a euthymic group. The Elderly Nutritional Index for Geriatric Malnutrition Assessment Bipolar Disorder Working Group also reported widespread bilateral patterns of reduced cortical thickness in the frontal, temporal, and parietal regions among 6,503 adults with BP (Hibar et al., 2018). These findings, along with our microimaging findings, converge to indicate that the ability to adjust the intrinsic emotional network is impaired in animal models of BP and in patients with this disorder.

Our finding that brain Ca^2+^ activity was related negatively only to the severity of depressive-like symptoms is supported by macro-imaging findings. For example, macro-imaging studies have documented correlations of depressive symptom severity with brain functional and structural alterations (Vai et al., 2019; Gong et al., 2020, 2021; Angelescu et al., 2021; Cahn et al., 2021; Keramatian et al., 2021). EEG studies have revealed correlations between brain neuron impairment and the severity of depressive symptoms (Banwari et al., 2015; Zhou et al., 2015; Liu et al., 2018; Belleau et al., 2019; Bhatt et al., 2021; Gao et al., 2021). We found no correlation between brain neuron impairment and the severity of manic-like symptoms, inconsistent with many previous findings (Dixon et al., 1994; Brody et al., 2003; Ettenberg et al., 2020; Gao et al., 2021), which may be related to the “double-hit” nature of our murine model. Further studies are needed to explore the precise pathological features of BP.

Clinical and macro-neuroimaging findings suggest that cognitive impairment is a core symptom in patients with BP; micro-neuroimaging studies have also revealed cognitive impairment in animal models of mania and depression (Banwari et al., 2015; Zhou et al., 2015; Hiser and Koenigs, 2018; Belleau et al., 2019; Mondimore, 2020; Harrison et al., 2021; Bhatt et al., 2021; Gao et al., 2021). Our data constitute new evidence for this phenomenon.

In summary, the use of *in vivo* two-photon imaging to examine a murine model of BP reveals brain neural activity patterns in the depressive and manic phases of this disorder (although the use of ketamine to induce the manic phase needs to be assessed further; the use of another method that does not cause brain neurophysiological alterations would be ideal). The consecutive induction of the depressive and manic phases in the same mice enables characterization of the trajectory of these patterns, providing information for the examination of phase switching in patients who experience antidepressant-induced mania. In turn, this characterization can form the basis for the examination of the effects of different treatment strategies in terms of improvement in brain activity and provide reference information for clinical practice. Although the translation of basic research findings to clinical practice is a lengthy process that is not guaranteed to succeed, translational research is needed to promote advances in therapeutics.

### Limitations

This study has several limitations. First, the model used in this study and others, in which ketamine is used to induce the manic phase, is valid, but the accuracy of this assessment has not been discussed in past years, although the aims of previous studies were to explore new methods for the improvement of treatment effects in patients with treatment-resistant depression and to rapidly reduce suicide attempts due to severe depressive symptoms in BP (Valvassori et al., 2019; Kim and Monteggia, 2020; McIntyre et al., 2020; Chen et al., 2021; Dean et al., 2021; Keilp et al., 2021; Magalhães et al., 2021; Nikayin and Sanacora, 2021; Wilkowska et al., 2021). A reviewer of this article led us to rethink the validity of the use of ketamine to induce the manic phase when studying BP (a physiopathological process), as drug-induced manic behavior may have different underlying mechanisms, Strictly speaking, a ketamine-induced manic phase can only represent such a phase induced by antidepressants, which is usually observed in clinical practice. The use of ketamine (including es-ketamine and dextroamphetamine, previously shown to be inferior for the treatment of depression) to treat serious depression may be better than other approaches, but its use to model mania in studies of BP-related brain features requires further examination. Ketamine causes brain neurophysiological alterations leading to mania-like behavior, therefore, its use to explore treatments targeting depressive symptoms is suitable, but we believe that model improvement is needed for applications. In future research, multiple strategies should be explored to fully represent the manic phase of BP. Although the findings of the present study have limited value, they provide a basis for further study. Second, our findings may be attributable to our induction of the manic phase immediately after the depressive phase in this study. An intervening interval of time may have allowed for brain recovery, avoiding a “double hit” effect of the CUMS and ketamine treatments. However, this dual-modeling approach has not been attempted in previous studies, and experience from clinical practice suggests that most patients with BP switch phases rapidly; some patients even have mixed manic/depressive episodes (Sharma et al., 2016). In the future, we will design and conduct multiple-arm studies to clarify this issue and identify a better method for BP modeling. Third, we used only a few behavioral and cognitive indices, although these indices are classic for animal models. The development of additional tests for the assessment of cognitive alterations might aid a more detailed description of the pathological mechanisms of BP.

## Conclusion

In this study, we observed brain Ca^2+^ activity in an animal model of BP with a chronic unpredictable mild stress–induced depressive phase and a subsequent antidepressant-induced manic phase. In the depressive phase, brain neural activity alterations correlated with depressive symptoms and cognitive impairment. In the manic phase, cognitive impairment, but not brain neural activity, correlated with manic symptoms. Findings from this animal study expand our understanding of phase-switching mechanisms in patients with BP, but further research is needed.

## Data Availability Statement

The datasets generated and analyzed during the present study are available from the corresponding author upon reasonable request.

## Ethics Statement

The study was approved by the Committee for Animal Care and Use of Tianjin Medical University Affiliated Tianjin Fourth Centre Hospital (IRB-no. 801758).

## Author Contributions

CZ and HT conceived and designed the research. MC, GC, GD, TF, ZC, LC, SC, CeC, JP, XL, ChC, JZ, FZ, and CL collected the data and conducted the research. MC, GC, HT, WY, XS, and CZ analyzed and interpreted the data. CZ and HT wrote the initial manuscript. WY and XS revised the manuscript. CZ had primary responsibility for the final manuscript content. All authors read and approved the final manuscript. All authors contributed to the article and approved the submitted version.

## Funding

This work was supported by grants from the National Natural Science Foundation of China (81871052 and 82171053 to CZ); National Key R&D Program of China (2016YFC1307000 to WY); the Key Projects of the Natural Science Foundation of Tianjin, China (17JCZDJC35700 to CZ); the Tianjin Health Bureau Foundation (2014KR02 to CZ); and the Tianjin Science and Technology Bureau (15JCYBJC50800 to HT).

## Conflict of Interest

The authors declare that the research was conducted in the absence of any commercial or financial relationships that could be construed as a potential conflict of interest.

## Publisher’s Note

All claims expressed in this article are solely those of the authors and do not necessarily represent those of their affiliated organizations, or those of the publisher, the editors and the reviewers. Any product that may be evaluated in this article, or claim that may be made by its manufacturer, is not guaranteed or endorsed by the publisher.

## Footnotes

^1^ https://www.nih.gov/about-nih/what-we-do/nih-almanac/national-institute-mental-health-nimh

## ABBREVIATIONS

BD: Bipolar disorder
CUMS: Chronic unpredictable mild stress
EEG: Electroencephalographic
MRI: Magnetic resonance imaging
PPI: Prepulse inhibition
PP: Prepulse
PA: Startle stimulus
ReHo: Regional homogeneity
TPC: Temporal cortex.
